# Participation amongst people ageing with neuromuscular disease: a qualitative study of lived experiences

**DOI:** 10.1002/nop2.966

**Published:** 2021-06-24

**Authors:** Louise Abildgaard Møller, Bente Martinsen, Ulla Werlauff, Pia Dreyer

**Affiliations:** ^1^ Research Department of Neurology Aarhus University Hospital Aarhus N Denmark; ^2^ Department of Public Health Section of Nursing Faculty of Health Aarhus University Copenhagen NV Denmark; ^3^ The Danish National Rehabilitation Center for Neuromuscular Diseases Aarhus Denmark; ^4^ Institute of Public Health Section of Nursing University of Aarhus Denmark; ^5^ Department of Intensive Care Aarhus University Hospital Aarhus N Denmark; ^6^ University of Bergen Bergen Norway

**Keywords:** Ageing, Hermeneutics, Narratives, Neuromuscular disease, Participation, Qualitative Research, Transitional Care

## Abstract

**Aim:**

To explore the lived experiences of participation in everyday life ageing with neuromuscular disease (NMD).

**Design:**

A qualitative study using a phenomenological‐hermeneutic approach.

**Methods:**

Data were gathered through interviews with 15 persons living with NMD in 2018. A three‐levelled analysis and interpretation influenced by Paul Ricoeur's philosophy were conducted.

The Consolidated Criteria for Reporting Qualitative Research (COREQ) checklist was used from May 2018 to December 2018.

**Results:**

Three themes were identified: “Endless adaptations change the fundamentals of everyday life ageing with NMD,” “The ‘swamp’ of deterioration” is traversed through experiences of belonging and relationship,” “Being disabled by a professional knowledge gap and stereotypical images.” In these themes, the experience of participation in everyday life ageing with NMD appeared to depend on the ability to adapt constantly. Through participation, a sense of belonging and purpose was maintained. Lack of knowledge amongst professionals may negatively affect the ongoing participation of people ageing with NMD.

## 1 INTRODUCTION

The term “everyday life” covers all human activities situated in and across a range of spaces and contexts such as home, work, educational institutions and recreational arenas (Schraube & Højholt, 2016). Engaging in everyday life is vital for humans (Law, 2002). Everyday life is where we make our worlds and where our worlds make us.

Persons living with neuromuscular disease (NMD) strive to live everyday life as others do and engage actively in everyday life (Boström & Ahlström, 2004). Advances in diagnostics and treatment of NMD‐related disabilities warrant focus on coherence in everyday life for people ageing with NMD (Gibson et al., 2009).

## 2 BACKGROUND

Neuromuscular disease is an overall term covering a wide range of incurable disorders affecting muscle function. The course of NMD subtypes varies. Some subtypes progress quickly, causing rapid decrease in physical function and comprehensive impairment. Other subtypes occur in adulthood and may progress more slowly (Aho et al., 2019; Meade et al., 2018). Life with NMD has been addressed as living with a chronic, deteriorating disease‐causing disabilities (Boström & Ahlström, 2004). Persons living with chronic disease and disability face two unique circumstances already in midlife. One is secondary health conditions, which are usually associated with ageing. The second is the phenomenon called “accelerated ageing” (Boström & Ahlström, 2004; Moll & Cott, 2013). Both circumstances occur when the functional symptoms of ageing are superimposed on existing disabilities. People ageing with disabilities experience symptoms of physical decline, usually related to later life, 10–20 years earlier than their able‐bodied counterparts, causing an acceleration of the ageing process (Groah et al., 2012; Klingbeil et al., 2004; Naidoo et al., 2012; Remillard et al., 2017). From an early point in midlife, the progression of NMD and ageing per se may cause accelerating weakness resulting in a complex situation with simultaneous need for age‐ and disease‐related care and rehabilitation (Martinsen & Dreyer, 2016; Rush et al., 2013). Ageing with disability has been identified as a highly vulnerable issue due to concerns of incoherent and complex care pathways (Martinsen & Dreyer, 2016; Yorkston et al., 2010). A central issue in ageing with NMD is fear of an unnecessarily passive life (Martinsen & Dreyer, 2016).

The ability to participate in everyday life and as a member of society is increasingly important and represents a key goal and vision for many stakeholders, including citizens with disabilities, disability advocacy organizations, rehabilitation providers, community organizations and policymakers (Foster & Walker, 2015). In 2001, the World Health Organization (WHO) made the concept of participation a fundamental part of the agenda of the International Classification of Functioning, Disability and Health framework (ICF‐model) (World Health Organization, 2001). Together with the four concepts, body functions and structures, activities, environmental factors and personal factors, participation forms the multi‐purpose tool, the ICF Model. This model is intended to conceptualize functioning as a “dynamic interaction between a person's health condition, environmental factors and personal factors” (World Health Organization, 2013). The WHO defined the concept of participation as “involvement in a life situation” (World Health Organization, 2001). This definition has been characterized as having fuzzy edges and lacking universal acceptance (Imms et al., 2016), which hampers operationalization of, for instance, care or rehabilitation initiatives aimed at participation improvements (Imms et al., 2016). Nevertheless, the role of participation has been repeatedly pointed out as important and vital in human life because participation leads to life satisfaction and personal development (Law, 2002; Martin Ginis et al., 2017).

It has been argued that the major issue in managing long‐term illness, such as NMD, is more likely to be social than medical (Boström & Ahlström, 2004), meaning that dealing with enduring illness encompasses the challenge of incorporating the illness into a coherent everyday life (Boström & Ahlström, 2004). In light of participation viewed as involvement in life situations (World Health Organization, 2001), knowledge from beyond medical settings focussing on how people experience involvement in everyday life is important. However, such knowledge is sparse, and this knowledge gap may affect the ability of health‐ and social care professionals to support coherent lives for people ageing with NMD. Thus, the objective of this study is to explore lived experiences of participation in everyday life ageing with NMD, from a firsthand perspective.

## 3 THE STUDY

### 3.1 Design

This study is a qualitative study, which from the theoretical perspective of phenomenological‐hermeneutics seeks to answer; How participation is experienced in everyday life when ageing with NMD? The theory of interpretation by the French philosopher Paul Ricoeur, (1976) inspired the analysis.

The COnsolidated criteria for REporting Qualitative Research (COREQ) checklist was used to secure accurate and complete reporting (Tong et al., 2007).

## 4 METHOD

### 4.1 Participants and data collection

This study is a part of a Danish project that broadly explores everyday life ageing with NMD, in which participation has emerged as a central phenomenon. Participants were recruited from both the Danish National Rehabilitation Centre for Neuromuscular Disease and the University Hospital of Aarhus, Denmark.

Sixteen persons were invited by email to take part in the study; 15 participants covering the five different regions of Denmark, eight women and seven men, joined the study. The participants were purposively sampled (Gentles & Vilches, 2017) with the aim of ensuring variation on different parameters potentially affecting the experience of ageing with NMD. The participants varied on the following parameters: age (range from 42–72 years), gender, disease onset, NMD subtype, ambulatory status, occupational status, living situation and need of care support. The range of necessary assistance varied from needing assistance around the clock to managing with little informal assistance. None of the participants was institutionalized. Inclusion criteria were as follows: 1) clinically confirmed NMD diagnosis, 2) ability to understand the information material and consent forms and 3) ≥40 years. Due to an applied life course perspective, the last inclusion criterion enabled going beyond contemporary understandings of ageing and look at ageing with respect to the individual's whole biography (Grassman & Whitaker, 2013).

The first author conducted the individual interviews starting with an introduction of professional background, commitment to the research field and the purpose of the study. A semi‐structured narrative approach was used (Brinkmann & Kvale, 2018) and an interview guide was developed and pilot tested. Central questions were; “Can you tell me, how a typical day was for you 10 years ago?” followed by “How is a typical day like for you now?” Probing questions were asked until the interviewer understood the participant's perspective (Saunders et al., 2018). By participant's choice, 12 interviews were at the participant's home and three by phone. The interviews lasted between 44 min and 2 hr and 45 min (18 hr of recorded speech and 374 pages of text). One of the participants needed the presence of an assistant due to respiratory status.

### 4.2 Methodological frame

Ricoeur's phenomenological‐hermeneutics and specifically the theory of interpretation was the theoretical inspiration due to the methodology's ability to describe and reach an in‐depth understanding (Ricoeur, 1973, 1976). Influenced by Ricoeur, the analysis moved dialectically from a “surface interpretation” (the naïve reading) to an in‐depth interpretation of the verbatim‐transcribed empirical material (the comprehensive understanding). The novel and comprehensive understanding reveals new facets of “being‐in‐the‐world” (Dreyer & Pedersen, 2009; Ricoeur, 1973).

Naïve reading is the first analytical step to grasp the understanding and meaning of the text. The interviews were read as one entire text in order to gain an initial impression of how participation is experienced. The naïve reading was guided by the touching elements in the text.

The structural analysis involves dialectic movements between the three levels in the text: *what is said –* quotations, *what the texts speaks about –* the meaning of the text and *theme(s)* (Dreyer & Pedersen, 2009; Ricoeur, 1976). The comprehensive understanding is achieved through distancing from “the inside world of the text” through discussion of the identified themes with “the world outside the text.” Concretely, the identified themes are critically discussed with respect to relevant theory on successful ageing and critical perspectives on this concept. The result of the critical discussion is the comprehensive understanding (Dreyer & Pedersen, 2009; Ricoeur, 1973).

The analysis was systematized using NVivo 11 software (QSR International, 2021; Zamawe, 2015) following the three steps in a Ricoeur‐inspired analysis: naïve reading, structural analysis and comprehensive understanding (Dreyer & Pedersen, 2009; Ricoeur, 1976). Dialectic movements in the analysis were compatible with the functions in NVivo. Movements between whole and parts in the analysis were enabled by the file function. Here the text figured as a whole. In the nodes‐function, the different parts (quotations) figured in different nodes. Memos were written, displaying what the text “spoke about”. These memos were organized under a theme title.

### 4.3 Rigour

With a phenomenological‐hermeneutic approach, an open‐minded, non‐judgmental attitude to the phenomenon being studied is essential (Lindseth & Norberg, 2004). Thus, continuous reflections on our preunderstandings of participation in everyday life ageing with NMD were part of the study. Each element of surprise served as a reminder of our preunderstandings. During the data collection, the first author let the preunderstanding as an experienced neurological nurse guide the interview situation. Members of the research team were likewise guided by their profession and area of expertise when interpreting the data.

According to Ricoeur, (1976), phenomenological‐hermeneutics is an argumentative discipline and this is important with respect to analytical rigour. The process of analysis and critical interpretation also consists of an ongoing dialectical movement between the naïve reading, structural analysis and critical interpretation and discussion (Simonÿ et al., 2018). It is in this movement that the suitability of the interpretation lies (Ricoeur, 1976, p. 78).

## 5 Ethical considerations

In accordance with the Helsinki Declaration and the ethical guidelines of the Nordic Nurses’ Federation (Snaedal, 2013), the participants received written and verbal information about the purpose of the study, anonymity and the opportunity to withdraw from the study. The participants had time to consider their participation before written consent was given. The study was approved by the Danish Data Protection Agency, journal number 1‐16‐02‐38‐18. According to the Danish law, Research Ethics Committee approval was not necessary. Guidelines for safe data management were followed. Conducting interviews is a moral practice in which the interviewer must be aware of the asymmetric relationship (Kvale, 2009). Therefore, the first author strove to adopt an attentive and humble attitude to avoid causing the participants any harm.

## 6 RESULTS

The naïve reading gave an intuitive understanding of the text based on what touched the readers. The initial understanding of participation in everyday life ageing with NMD was that participation is tailored to each individual lifeworld and is defined individually. Despite this individuality, being able to maintain participation is equally essential for the participants. Daily chores, family obligations, hobbies, being part of something and of use to someone are central elements of participation. Maintaining participation requires the use of different strategies; and in the scope of these strategies, the participants range from preparing as best and in as timely a fashion as possible to focus on living one day at a time. Participation in everyday life ageing with NMD is a complex and individualized matter.

### 6.1 Endless adaptations change the fundamentals of everyday life ageing with NMD

Changes related to age and NMD progression are experienced as being entangled. Differentiating between them is complicated due to the similarity in bodily manifestations of such changes. Intuitively, the participants attributed the exhaustion they experienced to disease progression. The gradual progression of NMD causes changes in everyday life to be adapted in an endless flow. An ongoing need to find new rhythms in everyday life arises, and the ongoing nature of the adaptations makes them invisible: “it goes so slowly that you do not quite realize it. So when people ask what and how much something has changed, there are things I haven't really noticed.” (I13). This invisibility results in even fundamental changes being adapted into everyday life without the appropriateness of the level of the adaptation being questioned:

If I am going out, I have to get my jacket on while I am helped to be dressed in the morning because I am no longer capable of getting my jacket on by putting it on myself. Otherwise, I would need to call for assistance from the health personnel from the municipality, but I think that would be stupid. So instead, I have to plan what I am doing for the entire day (I10).

Radical changes such as spending the day in outdoor clothes are adapted into the participants’ everyday lives in their pursuit of holding on to elementary elements of life, such as going outdoors. Adapting changes, even drastic changes, to everyday life is the participants’ strategy for maintaining the experience of independence and autonomy. Nevertheless, the independence and autonomy achieved through such adaptations may be hollow because the decisions left for the participants to make themselves are taken in relation to what the health and social care systems offer. Even though participants make choices of their own, these choices are made in a frame defined by the “system”. Thus, what might seem self‐made choices intended to facilitate independence and participation might lead to the opposite. Indeed, the narrowness of the frame in which apparently self‐made choices are made results in non‐participation and have the unintended result of increasing existing dependency. The illusion of “self‐made” choices blinds the participants to the role of adaptation. Every adaptation extends participants’ perceptions of what they consider reasonable adaptions. Combined with resignation, adaptations result in “over‐adaptations” taking place, causing participants to adjust and accept prerequisites for participation that may seem absurd to others. For instance, in pursuing participation, the rhythm of everyday life is adapted to the extent that several daytime hours are spent dressed in outdoor clothes in order to be able to participate in outdoor activities in the afternoon. Such adaptations seem a direct contradiction to participation.

Participants adapt to a mindset of prioritizing life situations they perceive as participation. These specific “everyday life tasks” play a crucial role in the participants’ perception of themselves and of what brings joy and meaning to their lives. These tasks are prioritized despite a statistically significant amount of time and energy being spent on carrying them out: “when I cook, I start preparing in the morning. I peel potatoes. It is difficult, so I begin with that. I peel eight to ten potatoes… It about takes 2 and a half to 3 hours to get them peeled.” Decreased performance speed does not change the perceived importance of these tasks or the participants’ determination to carry them out. A change in the flow and rhythm of everyday life can be accepted, and the rhythm of everyday life can be adapted if it allows the participants to continue carrying out tasks with value from their perspective. Endless adaptations are the strategy used to maintain participation through involvement in these individually defined tasks.

The importance of structured and controlled flow in everyday life is described by the participants. Even small changes may interrupt the vital structure of everyday life. When that happens, the foundation for participation is negatively affected. The need for stability is reflected in an increased need for control: “I’ve become more controlling, I think, and it's annoying and I’m not as spontaneous as before. It must be because everything is troublesome, and I struggle.” (I1). A strong need for control and perseverant effort to maintain flow and structure in everyday life is described as a matter of physical and mental wellbeing: “Having something to get up for in the morning and leaving my home means something to me, both mentally and physically… Being active loosens me up both mentally and physically.” (I5). In other words, the participants experience that structure in everyday life is a basic prerequisite for participation. Holding on to certain pivotal tasks structures everyday life and maintains the self‐image of being a human.

### 6.2 The “swamp” of deterioration is traversed through experiences of belonging and relationship

Hobbies serve several purposes in everyday life ageing with NMD. These purposes include making time pass and contributing statistically significant meaning and experiences of belonging. This is because the experience of having someone or something, for instance, a workplace or a hobby community, depending on you and demanding something of you is described as fundamental to the experiences of belonging. Furthermore, the achievement of meaning in life through participation is described. Challenges to participation are described with respect to ageing with NMD: “Some people are better at staying active and engaging in stuff, and I was good at that for many years. I engaged actively in associations and groups, but as the years went by, I couldn't manage anymore” (I13). Worries of potentially falling into a “swamp” of isolation and deterioration at the end of one's working life underline the meaning and sense of belonging achieved through participation: “I realize that the moment I am not in the job market anymore, I REALLY need to find something to be part of… I will get bogged down if I am not accountable to anyone but myself.” (I10). In addition to the risk of sinking into a state of indifference when the possibilities for participation are reduced, the issue of stigma comes into play. Descriptions of stigma and becoming a burden are related to descriptions of how society regards individuals who leave the workforce early or never enter the workforce:

it is not possible to sink lower as a person than being early retired. … You are not allowed to get a loan from the bank and you are looked on as someone who does not WISH to work. … I feel devalued to the point of having zero value, and on top of that I am ageing and becoming a burden to society – the burden of ageing. (I15)

Experiences of stigma and being a burden are counteracted through participation. Thus, engaging in hobbies, regardless of their specific character, is experienced as important and continues to be so despite the declining physical strength and restricted participation. Despite experiences of restricted participation, the experience of importance remains. Participating through hobbies causes the participants to have to prioritize. Due to the high amount of time and energy spent on appointments related to caring for the body, the participants experience having no time or energy left to engage in hobbies: “I spend a lot of time going to a physical therapist or the dentist, etc.…. Then someone has to come and help me shop or clean. I spend the entire week on such things, and when the weekend comes, I am totally exhausted.” (I4). Additionally, having to quit beloved hobbies due to physical deterioration, thus losing important and meaningful elements in everyday life is described. The sincere wish to stay engaged with hobbies, for instance, may also be due to the desire to belong in the community that is associated with the hobby:

I am part of a hunting consortium, so I could in principle just go along if I wanted to…. The day I say that I do not feel like it anymore ‐ when I do not LOVE to go hunting anymore, I will let go. As long as I still have the desire and drive but lack the energy, my answer will be, if someone asks if I have been hunting, no I haven't, I haven't had the time. (I15)

The described importance and desire to stay engaged in hobbies illustrates that despite the physical decline, people ageing with NMD strive to belong and live active, meaningful lives. Through participation, the participants experience belonging and sharing meaningful moments with others.

The importance of relationships with those assisting the participants ranges from them being pivotal to participation to only being a practical matter. For instance, having the same personal assistants employed for many years may be preferred due to the interaction and the relationship experienced:

they [the assistants] have to fit to where I am in my life. What I think of at my age is not similar to what a 20‐year‐old person thinks of. You have much more life experience and we also need to have something to tlk about. If there is nothing to talk about… I would not be able to endure that. One of my assistants also reads a lot of newspapers and we talk a lot about what we read. (I13)

Thus, relationships with the assistants can be experienced as an essential element for participation in everyday life ageing with NMD. However, a different perspective on the role of assistants towards participation is also described. Participants who have others supporting their participation in their everyday life, such as spouses, work or children, describe the assistants as a necessary practical resource for participation and not as essential for the social aspect of participation.

### 6.3 Being disabled by a professional knowledge gap and stereotypical images

Not being able to explain what everyday life ageing with NMD is like concerns the participants, and meeting professionals who lack understanding and knowledge of what living with NMD and increasing age is like results in experiences of powerlessness and incapacitation.

Additionally, the participants describe how stereotypical images of life with NMD are applied to individual situations: “when I say I have neuromuscular disease, the reaction is: well then, you probably cannot do anything.” (I1) and “immediately someone has to figure out how I should live my life and ensure coherence.” (I8) The stereotypical image comes to play in a reality where the effect of NMD on bodily functions is not necessarily immediately apparent: “my problem and frustration is this: people see me and say you're not sick, you are strong—and yes I am really strong, just not for very long at a time.” (I15). Taken together, stereotypical images of NMD, the invisible impact of NMD and professionals’ knowledge gap result in participants experiencing being taken “hostage” in their own lives. The subjective assessment of a professional who may lack knowledge of NMD is a source of frustration for the participants and makes them feel incapacitated. Skilled navigation in and communication with health and social care professionals are experienced as crucial to getting the required support, care and rehabilitation. Nevertheless, navigating and communicating in these systems are experienced as difficult. An unequal power relation exists between the participants and the support system around them. The system can carry on without providing assistance for participants, but participants cannot necessarily carry on their everyday life without support from “the system.” In some cases, the needed support is not allocated due to professionals’ lack of knowledge of NMD. This may create insecurity about how to maintain everyday participation. Additionally, participation is experienced by being in as good health as possible. Again, the importance of professionals with knowledge on NMD comes up. Getting specialized treatment is described as fundamental to one's ability to participate and to the continued desire to live. Optimal medical treatment is essential for participation. Furthermore, the need for support and guidance on how to cope with endless adaptations is described. Lack of care coordination across the life course is addressed by the participants as having an effect on their experience of continued and supported participation: ”I wish there had been some coordination and someone saying: I have been following this patient for ten years now and I see these effects, which we need to pay attention to. I miss having such a person.” (I11). Lack of professional support and information is specifically highlighted as a problem with respect to transition to the last phase of life, being old and not knowing when it ends. Seeking guidance and support on when the last phase begins is described as a lonely task where participants have limited information in terms of their own intuition or from personal assistants.

### 6.4 Overall comprehensive understanding

The overall comprehensive understanding is that striving to minimize loss in participation through ongoing adaptation is an underlying principle of everyday life ageing with NMD. Experiences of belonging are gained through participation. Experiencing belonging is the best weapon against deterioration, isolation, stigma and loss of purpose in life ageing with NMD. Lack of knowledge amongst professionals affects has the coordination of care and rehabilitation of persons ageing with NMD. A risk of being incapacitated by the unequal power relation between the professionals’ coordinating care and rehabilitation exists and may be of vital importance for participation as a weapon to maintain meaning and purpose in everyday life ageing with NMD.

## 7 CRITICAL INTERPRETATION AND DISCUSSION

The findings of this study illustrate that participation in everyday life ageing with NMD may be a basic prerequisite to be considered similar to the “active engagement” described by Rowe and Kahn in their definition of successful ageing (Rowe & Kahn, 1997). In light of Rowe and Kahn's definition, it is argued that active engagement in life is similar to the concept of participation as involvement in life situations (World Health Organization, 2001). Looking through the lens of Rowe and Kahn's concept of successful ageing three main components are included in the definition of successful ageing; low probability of disease and disease‐related disability, high cognitive and physical function capacity and active engagement with life (Rowe & Kahn, 1987, 1997). In this light, individuals ageing with NMD are in a position making it difficult to age successfully due to various degrees of effect on all of the three main components in successful ageing. Such position has been widely criticized (Minkler & Fadem, 2002; Molton & Yorkston, 2017). Contradictory views on ageing with chronic disease and disability are at play. For the disability system, ageing is a success, but for the ageing network, disability is a failure (Ansello, 2004, p. 4). These contradictory views may constitute a barrier to successful ageing for disabled persons. It may be argued that participation is essential for successful ageing viewed from the patient's perspective. The findings in the present study are consistent with the results of Molton and Yorkston, (2017) about the importance of adaptation, belonging and the need for professional knowledge. Adaptation may thus pave the way for experiencing autonomy which aligns with the ability to adapt to new circumstances as essential for ageing successfully with the experience of autonomy (Molton & Yorkston, 2017). Furthermore, this study's results indicate that experiences of belonging may be achieved through participation in hobbies, work, family obligations and everyday chores. This reflects the idea that social connectedness is a core element in successful ageing (Molton & Yorkston, 2017). The role and importance of professional knowledge when supporting continued participation in life ageing with NMD is accentuated in the results of this present study. This aligns with the importance of access to appropriate healthcare as a core element in successful ageing with disability (Molton & Yorkston, 2017).

In many ways, our findings on continuous adaptation as the foundation of participation and experiencing belonging through participation confirm the results of Molton and Yorkston, (2017) and Young et al. 2009 who advocate that successful ageing may be accomplished by people with chronic diseases through greater emphasis on psychological and/or social mechanisms that compensate for physiological decline. Our findings also resonate theoretically with core components of Flood´s nursing theory of successful ageing (Flood, 2005), suggesting that the findings concerning participation are mirrored in theory focussing on support in successful ageing.

In this light, participation is crucial to the experience of ageing successfully or, put in other words, meaning and the sense of personal fulfilment in everyday life when ageing with NMD is experienced through participation. Thus, whether successful ageing is an obtainable goal for people ageing with NMD is highly dependent on maintaining the experience of participation, and professionals’ knowledge is essential for maintaining participation in life ageing with a chronic disease.

### 7.1 Limitations

The findings mirror‐lived experiences of participation in everyday life when ageing with NMD but may have broader relevance, such as for those ageing with other chronic, deteriorating diseases or conditions affecting physical functions.

Phenomenological‐hermeneutics was the approach used in this study, and the results are contextual (Ricoeur, 1976). Thus, the specific context of NMD should be taken into consideration if the results are translated into another setting such as instance ageing with another chronic disabling disease.

## 8 CONCLUSION

The findings highlight that participation is essential for experiencing meaning and sense of purpose when ageing with NMD. Participation in everyday life is obtained and continued through adaptation, which makes continued experiences of belonging possible. It is vital that people ageing with NMD experience an ongoing sense of belonging and relationships as the physical functioning declines. Experiences of belonging counteract a tendency to face isolation and loss of purpose in life, and the experience of belonging may be maintained through continued participation in hobbies and/or daily chores. Experience of lack of knowledge amongst professionals may negatively affect the continued participation of people ageing with NMD. Knowledge of NMD and ageing with NMD amongst professionals is experienced as crucial for gaining the needed support through care and rehabilitation initiatives focussing on maintaining participation.

## CONFLICT OF INTEREST

No potential conflict of interest was reported by the authors.

## AUTHOR CONTRIBUTIONS

LA, BM, UW, PD: design; data collection, analysis and interpretation; drafting the manuscript or revising it critically and approval of the version to be published. The authors is responsible for all aspects of the work in ensuring the integrity of every part. Each author has participated sufficiently to take public responsibility for the content.

## DISCLAIMERS

The views expressed in this article are the authors own and not an official position of the institution or funders.

## DISCLOSURE OF RELATIONSHIPS AND ACTIVITIES

Mrs. Møller reports grants from The Danish Regions and the Danish Health Confederation, grants from The charity foundation, Jaschafonden, grants from The Vanføre Foundation, grants from Muskelsvindsfonden, the Danish neuromuscular patients’ association, during the conduct of the study.

## Supporting information

Supplementary Material

## Data Availability

The data that support the findings of this study are available on request from the corresponding author. The data are not publicly available due to privacy or ethical restrictions.
