# Supplementary material for: Participation amongst people ageing with neuromuscular disease: a qualitative study of lived experiences
Source: Nurs Open. 2021 Jun 24;9(6):2675–82. doi: 10.1002/nop2.966 (PMC9584473; doi:10.1002/nop2.966)
Supplement: Supplementary file 1 — Supplementary Material [file NOP2-9-2675-s001.docx]

**Supplementary File 1: COREQ checklist**

Domain 1: Research team and reflexivity.

Personal Characteristics

1. Interviewer/facilitator: Which author/s conducted the interview or focus group?

The first author (page 5 )

1. Credentials: What were the researcher’s credentials? E.g. PhD, MD

MScN, PhD (title page)

1. Occupation: What was their occupation at the time of the study?

Ph.D. student

Associate professor

Head of research

Professor

(title page)

1. Gender: Was the researcher male or female?

Female (title page)

1. Experience and training: What experience or training did the researcher have?

The first author has some experience as a research nurse

The three other authors are senior researchers

(page 4 + title page)

Relationship with participants

1. Relationship established: Was a relationship established prior to study commencement?

yes

1. Participant knowledge of the Interviewer: What did the participants know about the researcher? e.g. personal goals, reasons for doing the Research

All interviews began with an introduction of the first author who conducted the interviews and an introduction to the purpose of the study (page 5).

1. Interviewer characteristics: What characteristics were reported about the interviewer/facilitator? e.g. Bias, assumptions, reasons and interests in the research topic

In the introduction of the first author the participant were informed on the authors professional background and commitment to the field of research (p. 5)

Domain 2: study design (see page 4-7 in the main manuscript).

Theoretical framework

1. Methodological orientation and Theory: What methodological orientation was stated to underpin the study? e.g. grounded theory, discourse analysis, ethnography, phenomenology, content analysis, participant selection

phenomenological hermeneutic approach (page4-6)

1. Sampling: How were participants selected? e.g. purposive, convenience, consecutive, snowball

Purposively sampling were used guided by the aim of ensuring variation (page 4)

1. Method of approach: How were participants approached? e.g. face-to-face, telephone, mail, email

By mail for invitation and for interview 12 were face to face and three were by phone (page 4 )

1. Sample size: How many participants were in the study?

15 (page 4)

1. Non-participation: How many people refused to participate or dropped out? Reasons? Setting

One declined the invitation (page 4)

1. Setting of data collection: Where was the data collected? e.g. home, clinic, workplace

12 interviews were carried out in the participant’s home. Three were done by phone due to the participants busy schedules (page 5)

1. Presence of non-participants: Was anyone else present besides the participants and researchers?

In one study a personal assistant were within hearing distance due to the participant’s respiratory needs (page 5).

1. Description of sample What are the important characteristics of the sample? e.g. demographic data, date, Data collection

Being diagnosed with NMD and +40 years. Varying on various parameters described on page 4 (page 4)

1. Interview guide: Were questions, prompts, guides provided by the authors? Was it pilot tested?

The first author pilot tested the interview guide and. The interview guide were semistructured. Examples of questions are provided on page 5 (page 5).

1. Repeat interviews: Were repeat interviews carried out? If yes, how many?

None

1. Audio/visual recording: Did the research use audio or visual recording to collect the data?

Audio (page 5)

1. Field notes: Were field notes made during and/or after the interview or focus group?

No

1. Duration: What was the duration of the interviews or focus group?

44 min – 2 h 45min (page 5)

1. Data saturation Was data saturation discussed?

Not directly with that word but indirectly by giving information on the use of probing questions until understanding is reached

1. Transcripts returned: Were transcripts returned to participants for comment and/or correction?

No

Domain 3: analysis and findings

Data analysis

1. Number of data coders: How many data coders coded the data?

2

1. Description of the coding tree: Did authors provide a description of the coding tree?

No

1. Derivation of themes: Were themes identified in advance or derived from the data?

Arrived from the data

1. Software: What software, if applicable, was used to manage the data?

NVivo 11

1. Participant checking: Did participants provide feedback on the findings?

No

Reporting

1. Quotations presented: Were participant quotations presented to illustrate the themes / findings? Was each quotation identified? e.g. participant number

Yes (page 8-12)

1. Data and findings consistent: Was there consistency between the data presented and the findings?

Yes (page 8-12)

1. Clarity of major themes: Were major themes clearly presented in the findings?

Yes (page 7-10 )

1. Clarity of minor themes: Is there a description of diverse cases or discussion of minor themes?

No minor themes
